# Effects of Extra Virgin Olive Oil and Petrolatum on Skin Barrier Function and Microtopography

**DOI:** 10.3390/jcm14134675

**Published:** 2025-07-02

**Authors:** Ana Rubio-Santoyo, Raquel Sanabria-de la Torre, Trinidad Montero-Vílchez, María Sierra Girón-Prieto, Almudena Gómez-Farto, Salvador Arias-Santiago

**Affiliations:** 1 Medicine Department, School of Medicine, University of Granada, 18016 Granada, Spain; anarubiosan00@gmail.com (A.R.-S.); tmonterov@gmail.com (T.M.-V.); salvadorarias@ugr.es (S.A.-S.); 2 Biosanitary Research Institute of Granada (ibs.GRANADA), 18014 Granada, Spain; mariasierra@ugr.es; 3 Department of Biochemistry and Molecular Biology III and Immunology, University of Granada, 18071 Granada, Spain; 4 Dermatology Department, Virgen de las Nieves University Hospital, 18014 Granada, Spain; 5 Centro de Salud de Armilla, Servicio Andaluz de Salud, 18100 Granada, Spain; 6 Instituto de Investigación Biotecnológica, Farmacéutica y Medicamentos Huérfanos, S.L, 18016 Granada, Spain; almudenagomez@invesbiofarm.com; 7 Department of Pharmacy & Pharmaceutical Technology, Faculty of Pharmacy, University of Granada, 18071 Granada, Spain

**Keywords:** EVOO, petrolatum, skin barrier function, microtopography, stratum corneum

## Abstract

**Background/Objectives**: Natural oils are widely promoted and used around the world as part of skincare. Among them, extra virgin olive oil (EVOO) stands out for its broad range of organic compositions and well-known moisturizing properties. This study aimed to evaluate the effects of topically applied EVOO compared to petrolatum on skin barrier function (SBF) and microtopography. **Methods**: A within-person randomized clinical trial was conducted in healthy adult volunteers. EVOO and petrolatum were applied to defined areas on the volar forearm. Parameters related to the SBF, including stratum corneum hydration (SCH), transepidermal water loss (TEWL), temperature, and erythema, were assessed. The skin microtopography was evaluated through two approaches: (1) topographic parameters—surface roughness, desquamation, smoothness, and wrinkles; and (2) stratum corneum (SC) composition—corneocytes subtypes and the desquamation index (DI). The participants completed a tolerability questionnaire for each product. **Results**: A total of 54 participants (50% female; mean age: 28.57 ± 11.02 years) completed the study. Both EVOO and petrolatum significantly improved the SBF by increasing SCH and reducing erythema and skin temperature. Petrolatum additionally reduced TEWL. Regarding the skin microtopography, both products decreased the desquamation index and reduced the prevalence of mature corneocyte types (types 2–5). These effects were more pronounced with petrolatum. Notably, EVOO significantly increased the proportion of early-stage corneocytes (type 1). **Conclusions**: Both EVOO and petrolatum effectively enhanced the SBF and improved the microtopographic features of the skin. While petrolatum exerted a stronger occlusive effect by reducing TEWL and desquamation, EVOO uniquely promoted epidermal renewal by increasing epidermal turnover.

## 1. Introduction

Plant oils have long been used in dermatology and cosmetology due to their wide-ranging physiological benefits for the skin [1]. The topical application of plant oils can provide a protective barrier by exerting an occlusive effect, thereby enhancing skin hydration through a reduction in transepidermal water loss (TEWL) [2]. Unlike systemic treatments, topical formulations have the advantages of higher local bioavailability and reduced systemic exposure, making them ideal for targeted skin therapy [1,2].

Several plant-derived oils have been studied for their ability to support skin health [3]. In particular, olive oil (OO) is rich in monounsaturated fatty acids (especially oleic acid) and contains a complex profile of bioactive compounds, such as polyphenols, including phenolic alcohols, acids, flavonoids, lignans, and secoiridoids [4]. These phenolic compounds exhibit strong antioxidant, anti-inflammatory, and skin-regenerative properties, which could provide enhanced support to the skin barrier function (SBF) and reduce cutaneous inflammation [4,5]. Extra virgin OO (EVOO) obtained by cold-pressing retains a higher concentration of these bioactive molecules compared to refined oils, due to the absence of heat or chemical processing [5]. EVOO could potentially offer superior benefits for maintaining and restoring skin barrier integrity [4,6,7]. In clinical practice, especially in primary care [8], EVOO is commonly used for procedures, such as softening earwax prior to cerumen removal, managing chronic wounds, and preventing xerosis in geriatric patients [8,9]. Due to its anti-inflammatory properties, it is also occasionally used in conditions such as eczema, rosacea, and psoriasis [9]. Similarly, petrolatum is widely used for skin disorders, including postoperative wounds [10], irritant contact dermatitis [11], and chronic hand eczema [12]. The use of both substances is often guided by their accessibility and low cost, particularly in resource-limited settings [9,10]. However, despite their widespread traditional use and favorable compositions, there remains limited robust scientific evidence supporting their effects on the biophysical parameters of the SBF.

Therefore, the aim of this study is to investigate the effects of topically applied EVOO and petrolatum on the SBF and microtopography in healthy adult volunteers. By assessing the key indicators, such as TEWL, hydration, and surface texture, this research seeks to elucidate the dermatological potential of EVOO and support its evidence-based use in skin care and therapy.

## 2. Materials and Methods

### 2.1. Study Design

A controlled within-subject trial was conducted to evaluate changes in the SBF and microtopography following the topical application of the study products in healthy adult volunteers. This within-subject design was chosen to minimize interindividual variability by allowing each participant to serve as their own control.

### 2.2. Participants

#### 2.2.1. Recruitment

Participants were recruited from the Dermatology Department of Virgen de las Nieves University Hospital in Granada, Spain, between December 2023 and February 2024.

#### 2.2.2. Inclusion Criteria

- Age ≥ 18 years.
- No skin diseases.
- Signed informed consent provided.

#### 2.2.3. Exclusion Criteria

- Failure to meet any of the inclusion criteria.
- Use of systemic or topical treatments that could interfere with the SBF.
- Pregnancy or breastfeeding.
- Refusal or inability to sign the informed consent form.

#### 2.2.4. Sample Size Calculation

Assuming an alpha risk of 0.05 and a beta risk of 0.2 in a two-sided test, a total of 52 subjects were required to recognize a difference equal to or greater than 5 units in the main variable (TEWL), assuming a common standard deviation of 6 subjects.

### 2.3. Test Products and Application Procedure

The study area was located on the right volar forearm of each participant. Three adjacent 9 cm^2^ test sites (3 cm × 3 cm) were demarcated, with a 2 cm separation between them to prevent cross-contamination or interaction between products. Each site received one of the following treatments:

- Control: Clean skin.
- EVOO: 0.2 mL of cold-pressed organic EVOO (Rodríguez del Pozo^®^, Jaén, Spain).
- Comparator: 0.2 mL of pharmaceutical-grade white petrolatum (Vaseline Orravan^®^, Barcelona, Spain).

**Figure 1:**
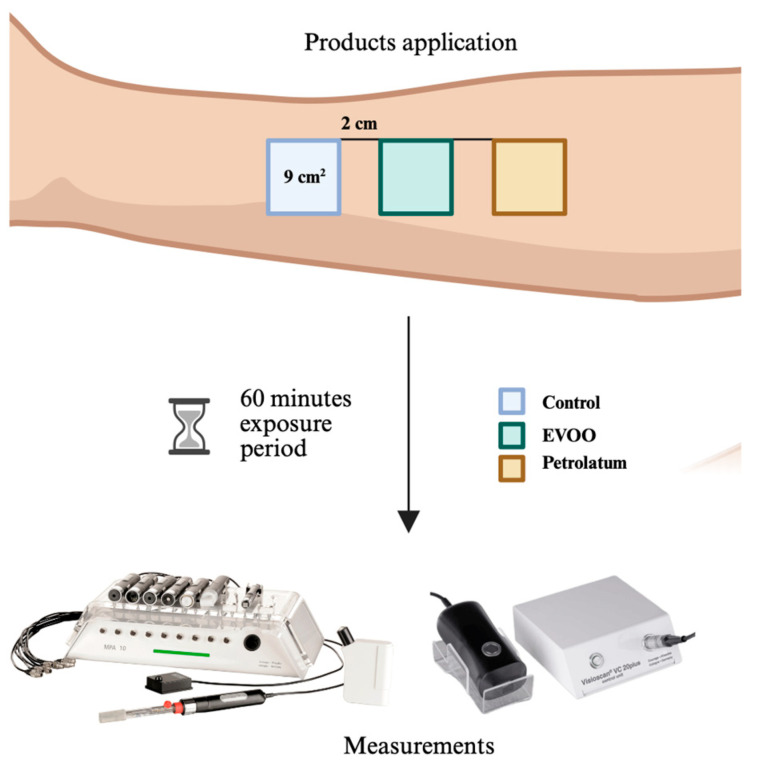
Data collection method.

The topical products were gently applied to the designated areas using a standardized protocol, ensuring that exactly 0.2 mL of each product was applied per site to maintain consistency. After application, a 60 min exposure period was observed. At the end of this period, any remaining product was gently blotted with a sterile gauze pad using a soft dabbing motion before biophysical measurements were taken (Figure 1).

### 2.4. Variables

The sociodemographic variables, such as age, sex, occupation, and skin care habits, were collected during a clinical interview. The SBF and microtopography measurements were performed in the same room with an average ambient humidity of 45.00% and a temperature of 22.00 ± 1.00 °C.

#### 2.4.1. Skin Barrier Function

- SCH (Corneometer^®^ CM 825, Microcaya^®^, Bilbao, Spain).
- TEWL (Tewameter^®^ TM 300, Microcaya^®^, Bilbao, Spain).
- pH (SkinpH-Meter^®^ PH 905, Microcaya^®^, Bilbao, Spain).
- Erythema and melanin index (Mexameter^®^ MX 18, Microcaya^®^, Bilbao, Spain).
- Skin temperature (Skin-Thermometer^®^ ST 500, Microcaya^®^, Bilbao, Spain).
- Skin surface lipids (Sebumeter^®^ SM 815, Microcaya^®^, Bilbao, Spain).
- Elasticity (Cutometer^®^ Dual MPA 580, Microcaya^®^, Bilbao, Spain):
  ▪ R0 or Uf (skin distensibility). The maximum displacement reached in a measurement.
  ▪ R2 or Ua/Uf (gross elasticity). The ratio of final retraction to maximal deformation. This value lies between 0 and 1; values closer to 1 are characteristic of more elastic skin.
  ▪ R7 or Ur/Uf (biological elasticity). The ratio between immediate retraction and total deformation. This parameter measures how much of the skin’s retraction happens right after the force is removed, compared to the total retraction observed throughout the entire measurement.

These parameters were measured with a multiprobe adapter (MPA^®^, Courage + Khazaka electronic GmbH, Köln, Germany). The measurements were taken 10 times on the volar forearm of each participant.

#### 2.4.2. Microtopography

##### Topography

- Smoothness (the evenness and texture of the skin’s surface);
- Roughness (the unevenness or irregularity of the skin’s surface);
- Desquamation (the process of shedding dead skin cells from the surface of the skin);
- Wrinkles (the presence and severity of fine lines and wrinkles on the skin);
- Surface (the size of the “wavy” surface is compared to the fully stretched flat (“ironed”) surface (x:1));
- Volume (the three-dimensional size or fullness of certain skin features, such as wrinkles or blemishes);
- Contrast (the difference in brightness, color, or texture between different areas of the skin);
- Entropy (the measurement of randomness or disorder in the skin’s texture or features) and variance (the degree of variation or difference in the skin’s texture or features);
- Energy (the intensity or strength of certain skin characteristics, such as pigmentation or hydration);
- Homogeneity (the uniformity or consistency of certain skin features across different areas);
- Anisotropy index (the measure of the directionality or orientation dependence of skin features, such as wrinkles or texture);
- Total cell count (the total number of cells present within the area of skin being analyzed).

These parameters were measured using a Visioscan^®^ VC 20plus (Courage + Khazaka electronic GmbH, Bilbao, Spain) and the SELS^®^ (Surface Evaluation of the Living Skin) method (Institute for Experimental Dermatology, Prof. Tronnier, University of Witten-Herdecke, Colonia, Germany).

##### Stratum Corneum Composition

- Desquamation index (quantifies the rate and extent of skin cell shedding: a high desquamation index indicates the presence of many large and thick corneocytes, which typically signifies dehydrated or damaged skin).
- Cell (corneocytes) types (both in % and mm^2^):
  - Class 1 cells (C1C): These are the newest cells, with minimal keratinization, appearing round and with intact structures.
  - Class 2 cells (C2C): Slightly older, these cells have increased keratinization and begin to show some structural changes.
  - Class 3 cells (C3C): More advanced in the keratinization process, these cells are flatter and display signs of lipid loss.
  - Class 4 cells (C4C): These mature cells are highly flattened with significant structural changes and have lost most of their intracellular components.
  - Class 5 cells (C5C): The oldest and most keratinized, these cells are extremely flat, often fragmented, and show extensive cellular breakdown.

These parameters were measured using Corneofix^®^ foil (Courage + Khazaka electronic GmbH, Bilbao, Spain) and Visioscan^®^ VC 20 plus equipment (Courage + Khazaka electronic GmbH, Bilbao, Spain).

### 2.5. Statistical Analyses

Descriptive analyses were conducted for all variables. Qualitative variables were expressed as proportions, while quantitative variables were presented as means with standard deviations. Normality of data was assessed using Kolmogorov–Smirnov and Shapiro–Wilk tests. Depending on variance homogeneity, evaluated via Levene’s test, Student’s *t*-test or Welch’s test was applied for comparing continuous variables between groups. Pearson’s correlation coefficient was used to examine relationships between normally distributed continuous variables. Statistical significance was set at *p* < 0.05. SPSS (Version 24.0) facilitated data analysis. G*Power 3.1.9.2, Heinrich-Heine-Universität Düsseldorf, was used to calculate sample size.

### 2.6. Ethics

This study was conducted in accordance with the guidelines of the Declaration of Helsinki and was approved by the Ethics Committee of Virgen de las Nieves University Hospital on 31 January 2024 (1877-N-23). The nature of the study was explained to all the participants, who agreed to participate by verbal and written consent. All measurements were noninvasive and participant data were kept confidential.

## 3. Results

### 3.1. Population Socio-Demographics

The study included 54 individuals, comprising 50.00% female (27/54), with a mean age of 28.57 years (±11.02). Among them, 24.10% (13/54) had a smoking habit and 66.70% (36/54) had an alcohol habit (Table 1).

### 3.2. Skin Barrier Function After Application of Extra Virgin Olive Oil and Petrolatum

**Figure 2:**
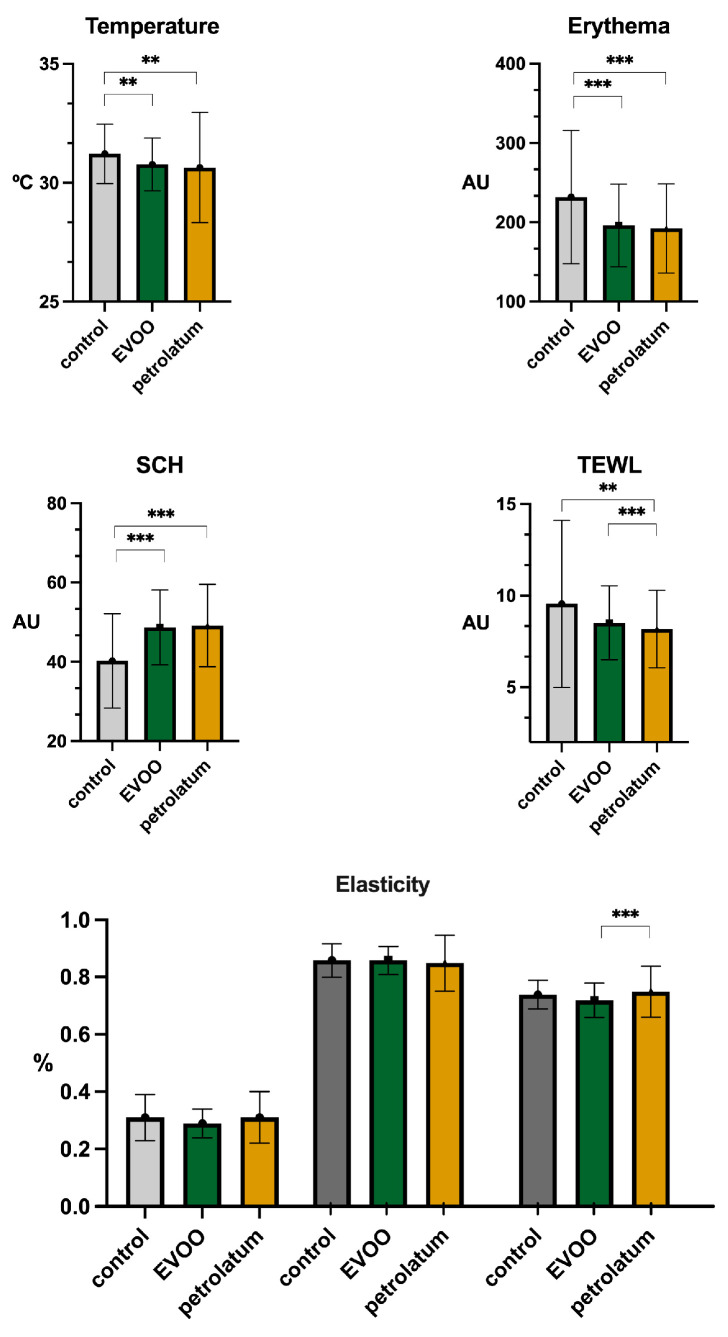
Biophysical parameters of skin measured at control, EVOO, and petrolatum sites. Elasticity is represented as R0 (**left**), R2 (**middle**), and R7 (**right**). “**” = *p* < 0.01; “***” = *p* < 0.001. Abbreviations: AU: arbitrary units; SCH: skin corneum hydration; TEWL: transepidermal water loss; EVOO: extra virgin olive oil.

The differences observed in the parameters measured with the MPA system are presented in Figure 2. The skin temperature and erythema significantly decreased following the application of both EVOO (31.22 °C vs. 30.77 °C, *p* < 0.01; 231.79 AU vs. 196.03 AU, *p* < 0.001) and petrolatum (31.22 °C vs. 30.64 °C, *p* = 0.014; 231.79 AU vs. 192.13 AU, *p* < 0.01). The SCH increased after the application of EVOO (40.23 AU vs. 48.66 AU, *p* < 0.001) and petrolatum (40.23 AU vs. 49.15 AU, *p* < 0.01). The TEWL showed a decreasing trend following treatment with EVOO (9.56 g·m^−2^·h^−1^ vs. 8.52 g·m^−2^·h^−1^, *p* = 0.06) and a significant decrease with petrolatum (9.56 g·m^−2^·h^−1^ vs. 8.18 g·m^−2^·h^−1^, *p* = 0.016). Furthermore, petrolatum produced a significant change in the TEWL compared to that of EVOO (8.52 g·m^−2^·h^−1^ vs. 8.18 g·m^−2^·h^−1^, *p* < 0.001). The R7 elasticity increased slightly after petrolatum application in comparison to EVOO (0.73% vs. 0.76%, *p* = 0.045). No other significant differences were found for the remaining parameters (Table 2).

### 3.3. Microtopography After Application of Extra Virgin Olive Oil and Petrolatum

#### 3.3.1. Surface Evaluation of Living Skin

The SELS parameters are shown in Table 3. The skin surface area increased by 72.38% after EVOO application (759.59% vs. 831.97%, *p* = 0.002) and by 95.15% after petrolatum use (759.59% vs. 854.75%, *p* < 0.01). The contrast also increased following both EVOO and petrolatum (1.57 AU vs. 1.83 AU, *p* = 0.02 and 1.57 AU vs. 1.91 AU, *p* < 0.001, respectively). The other variables, such as the variance, energy, and anisotropy index, changed significantly only after petrolatum application (5.45 AU vs. 5.79 AU, *p* = 0.2; 0.02 AU vs. 0.01 AU, *p* = 0.033; 28.85 AU vs. 22.95 AU, *p* < 0.001). The homogeneity decreased after both EVOO and petrolatum application (1.31 AU vs. 1.28 AU, *p* = 0.001; 1.31 AU vs. 1.27 AU, *p* < 0.001). The total number of corneocytes decreased significantly after EVOO application (130.74 AU vs. 106.92 AU, *p* < 0.001) and after petrolatum use (130.74 AU vs. 105.66 AU, *p* < 0.001). No other significant changes were observed in the remaining parameters.

#### 3.3.2. Desquamation

**Figure 3:**
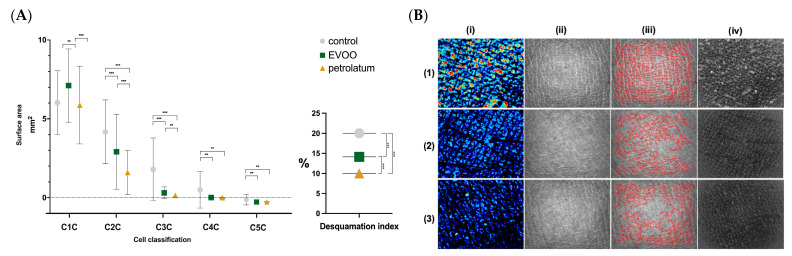
(**A**) The cellular composition of the stratum corneum and desquamation index in the control, EVOO-treated, and petrolatum-treated areas. “**” = *p* < 0.01; “***” = *p* < 0.001. C1C = class 1 cells, C2C = class 2 cells, C3C = class 3 cells, C4C = class 4 cells, C5C = class 5 cells. (**B**) Images captured with Visioscan^®^ VC 20 plus (Courage + Khazaka electronic GmbH, Bilbao, Spain). (**1**) Control skin, (**2**) skin after EVOO application, (**3**) skin after petrolatum application. (**i**) The desquamation was measured using Corneofix^®^ (Courage + Khazaka electronic GmbH, Bilbao, Spain). The corneocytes were analyzed by their thickness across five layers: very thick (red), still bright (orange), medium thick (green), less thick (light and dark blue), and the bottom layer (black). (**ii**) SELS evaluation. The images display a range of gray levels: the darker areas represent lines and wrinkles, while the extremely bright areas correspond to plateaus in the skin microrelief. (**iii**) This section shows the distribution of lines in the image structure, their directions, and the cells surrounded by lines (polygons). The distribution of red lines was taken into account for the anisotropy index calculation using Visioscan^®^ VC 20 plus software (Institute for Experimental Dermatology, Prof. Tronnier, University of Witten-Herdecke, Colonia, Germany). The higher the anisotropy index, the more directional the skin. Young skin should have a lower anisotropy index (many different directions) than older skin (more prominent directions). (**iv**) Irregular skin surface photographs were taken into account for the calculation of the texture parameters (contrast, entropy, variance, energy, and homogeneity) with Visioscan^®^ VC 20 plus software (Institute for Experimental Dermatology, Prof. Tronnier, University of Witten-Herdecke, Colonia, Germany).

The desquamation index and the cellular composition of the SC were both significantly affected by the application of EVOO and petrolatum, as shown in Figure 3.

The area of C1C on the SC was significantly higher after EVOO application compared to both the control and petrolatum-treated sites (7.11 mm^2^ vs 6.02 mm^2^, *p* = 0.012; 7.11 mm^2^ vs. 5.87 mm^2^, *p* < 0.001). The C2C and C3C decreased after both treatments compared to those of the control (4.19 mm^2^ vs. 2.92mm^2^, *p* = 0.001; 4.19mm^2^ vs. 1.61mm^2^, *p* < 0.001; 1.80 mm^2^ vs. 0.31 mm^2^, *p* < 0.001; 1.80 mm^2^ vs. 0.15 mm^2^, *p* < 0.001). The reductions in the C2C and C3C were significantly greater with petrolatum than with EVOO (2.92mm^2^ vs. 1.61mm^2^, *p* < 0.001; 0.31mm^2^ vs. 0.15mm^2^, *p* = 0.003). The C4C and C5C also decreased significantly after both EVOO and petrolatum use (0.51mm^2^ vs. 0.02mm^2^, *p* = 0.003; 0.51mm^2^ vs. 0.17mm^2^, *p* = 0.003; 0.15 mm^2^ vs. 0.004 mm^2^, *p* = 0.002; 0.15 mm^2^ vs. 0.005 mm^2^, *p* = 0.003). The desquamation index decreased significantly after EVOO application (20.03AU vs 14.13 AU, *p* < 0.001), and even more markedly with petrolatum, both compared to the control and to the EVOO site (20.03 AU vs. 10.03 AU, *p* < 0.001; 14.13 AU vs. 10.03 AU, *p* < 0.001) (Table 4).

### 3.4. Tolerability

Color scored higher after the use of EVOO compared to petrolatum, with an increase of 1.09 points (8.14 points vs. 7.05 points, *p* = 0.02). The rest of the tolerability parameters did not show significant differences (Table 5).

## 4. Discussion

The results obtained in this study demonstrate that both EVOO and petrolatum enhance the SBF, but with notable differences in their observed effects and potential mechanisms of action.

When the SBF is compromised, as seen in conditions like atopic dermatitis or psoriasis, the skin temperature, erythema, and TEWL increase [13,14,15], while the SCH decreases [16]. Both actives effectively reduced the temperature and erythema and improved the SCH. Petrolatum also produced a significant reduction in the TEWL [17]. In fact, petrolatum is widely recognized for its superior occlusive properties, which form a semi-permeable film. This occlusive ability of petrolatum also resulted in a slight improvement in skin elasticity, which may be related to the increased water retention [18]. Its barrier-forming capacity makes petrolatum especially beneficial in clinical contexts involving fissures, xerosis, and chronic wounds, where protection from external irritants and TEWL is crucial [10,11,12]. These findings align with previous studies on skin barrier excipients [19].

In our study, petrolatum induced more significant changes in the anisotropy and homogeneity, suggesting that its use leads to more uniform skin [20]. Furthermore, petrolatum reduced the number of corneocytes in the superficial layers, particularly those involved in desquamation (C2C, C3C, C4C, and C5C), resulting in a significantly lower desquamation index. Its hydrophobic nature created a physical barrier that prevented TEWL, thereby maintaining SCH and improving barrier integrity [21].

In contrast, EVOO exhibited a different effect. While it also improved the SBF parameters, EVOO showed a distinct pattern in the cellular composition of the SC. Unlike petrolatum, EVOO favored an increase in C1C, which are primarily involved in cell turnover and skin recovery. This suggests that EVOO may stimulate cellular regeneration. The observed increase in C1C following EVOO application may be attributed to its unique biochemical composition, particularly its phenolic compounds, unsaturated fatty acids, and antioxidant capacity [22]. EVOO is rich in bioactive components, such as hydroxytyrosol, oleuropein, and tyrosol, which have been shown to exert anti-inflammatory, antioxidant, and cytoprotective effects on skin cells [23].

These phenolic antioxidants may play a key role in supporting keratinocyte health and proliferation, protecting basal and suprabasal cells from oxidative damage, and promoting more effective epidermal turnover. In addition, oleic acid, the predominant fatty acid in EVOO, is known to enhance skin permeability and can facilitate the penetration of active compounds into the epidermis, potentially triggering biological responses at the level of keratinocyte differentiation and maturation [24].

The increase in C1C, which represent early-stage corneocytes, suggests that EVOO may promote a faster transition from viable keratinocytes to corneocytes, thereby accelerating desquamation and cellular renewal. This regenerative effect could be particularly advantageous in conditions requiring the stimulation of skin repair or turnover, such as post-inflammatory skin or aging skin [25,26]. The larger area of C1C observed after EVOO application could indicate an acceleration of the desquamation process and skin renewal, which may be beneficial in contexts where promoting skin regeneration is desired. This regenerative potential is supported by the clinical use of EVOO in the treatment of pressure ulcers [27], where it has been shown to promote wound healing, improve skin integrity, and reduce the incidence and severity of ulcers, particularly in immobilized or elderly patients [28].

The tolerance to both products was similar, with no significant differences in the evaluated characteristics, except for color, for which the participants reported a higher tolerance for the EVOO.

While both treatments improved the SBF, the effect of petrolatum appeared more pronounced in preventing TEWL, whereas EVOO seemed to have a more dynamic effect by promoting cell turnover.

The main limitation of this study is its cross-sectional design, which does not reflect the effects of continuous or long-term use of the moisturizing agents. This limits the ability to comprehensively evaluate the outcomes of daily use. Additionally, the assessment of the treatment effects was limited to a single time point (60 min post-application). Although this time frame is widely used in dermatological assessments and is considered appropriate for assessing immediate topical responses [29,30,31,32], it does not provide insight into the persistence of these effects over time. Nevertheless, a major strength of this study is the objective evaluation of the immediate effects on the skin barrier function and microtopography, using measurement techniques not previously applied in this context. Assessing the acute effects is particularly relevant for identifying rapid improvements, as well as potential irritation or early signs of sensitivity, which are key for the short-term safety and tolerability of topical formulations. This approach could help tailor the use of emollients to address the most affected parameters of barrier function, which may be particularly useful in treating skin conditions where these parameters are compromised. Furthermore, these findings can aid in the formulation of clinically relevant products adapted to specific skin needs. Future studies should include longer follow-up periods and repeated applications to better understand the long-term and cumulative impact of these formulations on skin barrier function and structure.

## 5. Conclusions

Both products enhanced the SBF and skin microtopography through different mechanisms. The biophysical parameters, such as temperature, erythema, SCH, and SC composition, improved with both actives. However, petrolatum demonstrated additional benefits, significantly improving TEWL and the desquamation index compared to EVOO. On the other hand, EVOO was particularly effective at enhancing C1C corneocytes, which are involved in cellular renewal and early-stage desquamation, suggesting its role in promoting skin regeneration.

## Figures and Tables

**Table 1:** Socio-demographic characteristics.

Variables	n = 54
Sex	
Male	27/54 (50.00%)
Female	27/54 (50.00%)
Age (years)	28.57 ± 11.02
Height (cm)	170.43 ± 8.44
Weight (kg)	69.41 ± 14.75
Civil Status	
Single	47/54 (87.00%)
Married	5/54 (9.30%)
Divorced	1/54 (1.90%)
Widowed	1/54 (1.90%)
Level of Study	
Basic	4/54 (7.40%)
Intermediate	11/54 (20.40%)
Higher	39/54 (72.20%)
Phototype	
I	0/54 (0.00%)
II	10/54 (18.50%)
III	36/54 (66.70%)
IV	7/54 (13.00%)
V	1/54 (1.90%)
VI	0/54 (0.00%)
Smoking Habit	
Yes	13/54 (24.10)
No	41/54 (75.90)
Alcohol Habit	
Yes	36/54 (66.70)
No	18/54 (33.30)
SDU/Week	2.48 ± 5.93
Personal History of Any Disease	
Yes	23/54 (42.60%)
No	31/54 (57.40%)
Family History of Diseases	
Yes	25/54 (46.30%)
No	29/54 (53.70%)
Family History of Dermatologic Disease	
Yes	14/54 (25.90%)
No	40/54 (74.10%)
Weekly Body Moisturizing (days/week)	2.09 ± 2.67
Weekly Facial Moisturizing (days/week)	3.94 ± 3.13
Use of Other Topical Skincare Products (non-medicated)	
Yes	15/54 (27.80%)
No	39/54 (72.20%)
Frequent Sun Exposure	
Yes	15/54 (27.80%)
No	39/54 (72.20%)
Sun Exposure (hours/week)	5.39 ± 9.58
Use of Sun Protection	
Yes	29/54 (53.70%)
No	25/54 (46.30%)
Medication	
Yes	17/54 (31.50%)
No	37/54 (68.50%)

For the quantitative measures, the mean and standard deviation (±) are used, and for the qualitative measures, the number (n/N) and percentage are used. “Use of other non-medicated topical skincare products” includes products not captured in other specific rows (e.g., cosmetic oils, cleansing balms, toners). SDU = standard drink unit, equivalent to 10 g of pure alcohol.

**Table 2:** Biophysical parameters of skin barrier function.

	Control	EVOO	Petrolatum	Differences: Control vs. EVOO	Differences: Control vs. Petrolatum	Differences: EVOO vs. Petrolatum	P1	P2	P3
**Temperature (°C)**	31.22 ± 1.25	30.77 ± 1.11	30.64 ± 2.32	0.50 ± 0.68	0.63 ± 1.82	0.13 ± 1.84	**<0.01**	**0.01**	0.60
**Melanine (AU)**	171.74 ± 51.95	165.71 ± 43.09	165.82 ± 40.9	6.03 ± 31.56	5.91 ± 33.19	−0.11 ± 15.30	0.16	0.19	0.95
**Erythema (AU)**	231.79 ± 84.17	196.03 ± 51.98	192.13 ± 56.12	35.75 ± 72.79	39.66 ± 72.8	3.90 ± 21.90	**<0.001**	**<0.001**	0.19
**SCH (AU)**	40.23 ± 11.89	48.66 ± 9.45	49.15 ± 10.4	−8.43 ± 10.10	−8.92 ± 12.2	−0.48 ± 9.64	**<0.001**	**<0.001**	0.71
**TEWL (g·m** **^−^** **^2^** **·h** **^−^** **^1^** **)**	9.56 ± 4.56	8.52 ± 2.02	8.18 ± 2.11	1.03 ± 4.02	1.37 ± 4.06	0.33 ± 0.63	0.06	**0.01**	**<0.001**
**R0 Elasticity (%)**	0.31 ± 0.08	0.29 ± 0.06	0.31 ± 0.05	0.01 ± 0.09	0.004 ± 0.08	−0.01 ± 0.05	0.25	0.72	0.19
**R2 Elasticity (%)**	0.87 ± 0.05	0.87 ± 0.05	0.86 ± 0.06	0.001 ± 0.05	0.01 ± 0.05	0.01 ± 0.05	0.90	0.2	0.19
**R7 Elasticity (%)**	0.75 ± 0.09	0.73 ± 0.10	0.76 ± 0.09	0.02 ± 0.1	−0.01 ± 0.09	−0.02 ± 0.10	0.10	0.70	**0.04**

The values for the control, EVOO, and petrolatum are presented as the mean and standard deviation (±). The *p*-values are based on Student’s *t*-test for paired sample comparisons. P1 corresponds to the comparison of the values for the control skin and the skin after application of EVOO; P2 corresponds to the comparison of the control skin and the skin after application of petrolatum. P3 corresponds to the comparison of the skin after applying EVOO and the skin after applying petrolatum. Abbreviations: AU: arbitrary units; SCH: skin corneum hydration; TEWL: transepidermal water loss; EVOO: extra virgin olive oil. Bold indicates statistically significant *p*-values (*p* < 0.05).

**Table 3:** Surface evaluation of living skin (SELS^®^).

	Control	EVOO	Petrolatum	Differences: Control vs. EVOO	Differences: Control vs. Petrolatum	Differences: EVOO vs. Petrolatum	P1	P2	P3
**Roughness (Ser)**	2.52 ± 1.65	2.49 ± 1.94	2.25 ± 1.38	0.02 ± 1.42	0.27 ± 1.55	0.24 ± 1.92	0.90	0.20	0.34
**Desquamation (Sesc)**	0.52 ± 0.69	0.53 ± 0.62	1.22 ± 3.57	−0.003 ± 0.7	−0.69 ± 3.52	−0.69 ± 3.52	0.97	0.15	0.15
**Smoothness (Sesm)**	218.13 ± 57.76	212.97 ± 55.04	215.23 ± 72.48	5.15 ± 71.23	2.89 ± 74.20	−2.26 ± 70.70	0.59	0.77	0.81
**Wrinkles (Sew)**	73.17 ± 97.08	53.97 ± 17.22	53.39 ± 13.8	19.19 ± 99.13	19.77 ± 98.01	0.57 ± 17.67	0.16	0.14	0.81
**Surface (%)**	759.59 ± 169.56	831.97 ± 148.27	854.75 ± 167.55	−72.38 ± 166.71	−95.15 ± 159.54	−22.77 ± 128.42	**0.002**	**<0.001**	0.19
**Volume (mm^3^)**	97.81 ± 22.25	99.21 ± 19.37	100.44 ± 17.39	−1.40 ± 28.49	−2.62 ± 21.88	−1.22 ± 22.05	0.72	0.38	0.68
**Contrast (AU)**	1.57 ± 0.57	1.83 ± 0.70	1.91 ± 0.75	−0.25 ± 0.56	−0.34 ± 0.57	−0.08 ± 0.49	**0.002**	**<0.001**	0.19
**Entropy (AU)**	4.12 ± 19.53	1.45 ± 0.04	1.44 ± 0.05	2.67 ± 19.53	2.67 ± 19.54	0.01 ± 0.03	0.32	0.32	0.24
**Variance (AU)**	5.45 ± 1.20	5.79 ± 1.40	5.96 ± 1.61	−0.34 ± 1.39	−0.50 ± 1.38	−0.16 ± 1.49	0.07	**0.01**	0.41
**Energy (AU)**	0.02 ± 0.01	0.01 ± 0.01	0.01 ± 0.01	0.001 ± 0.008	0.002 ± 0.01	0.0003 ± 0.01	0.13	**0.03**	0.67
**Homogeneity (AU)**	1.31 ± 0.08	1.28 ± 0.07	1.27 ± 0.08	0.03 ± 0.07	0.04 ± 0.08	0.01 ± 0.07	**0.001**	**<0.001**	0.32
**Anisotropy Index (AU)**	28.85 ± 11.77	25.38 ± 9.67	22.95 ± 8.60	3.46 ± 12.58	5.89 ± 11.19	2.42 ± 9.36	0.48	**<0.001**	0.63
**Total Cells (AU)**	130.74 ± 34.03	106.92 ± 22.52	105.66 ± 19.62	23.81 ± 36.84	25.07 ± 38.15	1.25 ± 26.05	**<0.001**	**<0.001**	0.72

The values for the control, EVOO, and petrolatum are calculated as the mean and standard deviation (±). Contrast: difference between the gray levels of two neighboring pixels; entropy: “disorder” of an image; variance: average of a local variance over a number of pixels; energy: rate of change in color/brightness/magnitude; homogeneity: uniformity of an image; anisotropy: describes the direction dependence of a material (the opposite of isotropy). The *p*-values are based on Student’s *t*-test for paired sample comparison. P1 corresponds to the comparison between the values for the control skin and the skin after application of EVOO; P2 corresponds to the comparison between the control skin and the skin after application of petrolatum. P3 corresponds to the comparison between the skin after applying EVOO and the skin after applying petrolatum. Abbreviations: AU: arbitrary units; EVOO: extra virgin olive oil. Bold indicates statistically significant *p*-values (*p* < 0.05).

**Table 4:** Stratum corneum cell counts and desquamation.

	Control	EVOO	Petrolatum	Differences: Control vs. EVOO	Differences: Control vs. Petrolatum	Differences: EVOO vs. Petrolatum	P1	P2	P3
**C1C (%)**	20.88 ± 7.03	24.7 ± 8.14	20.38 ± 8.54	−3.82 ± 10.79	0.49 ± 11.53	4.31 ± 8.30	**0.01**	0.75	**<0.001**
**C1C (mm^2^)**	6.02 ± 2.03	7.11 ± 2.34	5.87 ± 2.46	−1.09 ± 3.1	0.14 ± 3.31	1.23 ± 2.38	**0.01**	0.75	**<0.001**
**C2C (%)**	14.54 ± 8.59	10.16 ± 8.28	5.61 ± 4.88	4.37 ± 9.31	8.92 ± 8.92	4.55 ± 7.67	**<0.001**	**<0.001**	**<0.001**
**C2C (mm^2^)**	4.19 ± 2.51	2.92 ± 2.38	1.61 ± 1.40	1.27 ± 2.72	2.58 ± 2.61	1.30 ± 2.20	**<0.001**	**<0.001**	**<0.001**
**C3C (%)**	6.26 ± 7.54	1.09 ± 1.33	0.70 ± 1.60	5.16 ± 7.21	5.55 ± 7.44	0.38 ± 1.63	**<0.001**	**<0.001**	0.08
**C3C (mm^2^)**	1.80 ± 2.17	0.31 ± 0.38	0.15 ± 0.17	1.49 ± 2.07	1.65 ± 2.12	0.16 ± 0.38	**<0.001**	**<0.001**	**0.003**
**C4C (%)**	1.25 ± 2.19	0.07 ± 0.10	0.06 ± 0.12	1.17 ± 2.20	1.18 ± 2.2	0.01 ± 2.20	**<0.001**	**<0.001**	0.70
**C4C (mm^2^)**	0.51 ± 1.16	0.02 ± 0.03	0.17 ± 0.03	0.49 ± 1.16	0.49 ± 1.15	0.003 ± 0.04	**0.003**	**0.003**	0.57
**C5C (%)**	0.36 ± 0.63	0.02 ± 0.03	0.01 ± 0.03	0.34 ± 0.63	0.34 ± 0.63	−0.001 ± 0.04	**<0.001**	**<0.001**	0.92
**C5C (mm^2^)**	0.15 ± 0.33	0.004 ± 0.01	0.01 ± 0.01	0.14 ± 0.33	0.14 ± 0.33	−0.001 ± 0.01	**0.002**	**0.003**	0.62
**DI (AU)**	20.03 ± 10.15	14.13 ± 6.86	10.03 ± 5.15	5.90 ± 9.37	10.00 ± 8.44	4.09 ± 6.22	**<0.001**	**<0.001**	**<0.001**

The values for the control, EVOO, and petrolatum are calculated as the mean and standard deviation (±). The class 1 cells are the youngest corneocytes, indicating living tissue, while the class 5 cells are the oldest, indicating further desquamation. Class 2-4 corneocytes show the aging process from the initial stage (class 1) to the final stage (class 5). The area covered by corneocytes in the image is indicated in mm^2^ and in %. The desquamation index is calculated from the distribution of the different classes of corneocytes, where the thicker scales (class 5) have a higher weight in the formula than the thinner layers (class 1). The *p*-values are based on Student’s *t*-test for paired sample comparison. P1 corresponds to the comparison between the values for the control skin and the skin after application of EVOO; P2 corresponds to the comparison between the control skin and the skin after application of petrolatum. P3 corresponds to the comparison between the skin after applying EVOO and the skin after applying petrolatum. C1C: class 1 cells; C2C: class 2 cells; C3C: class 3 cells; C4C: class 4 cells; C5C: class 5 cells; DI: desquamation index; EVOO: extra virgin olive oil; AU: arbitrary units. Bold indicates statistically significant *p*-values (*p* < 0.05).

**Table 5:** Tolerability parameters’ scores after application of EVOO and petrolatum.

	EVOO	Petrolatum	Differences: EVOO vs. Petrolatum	*p*
**Color**	8.14 ± 2.45	7.05 ± 2.94	1.09 ± 2.94	**0.02**
**Odor**	7.59 ± 2.77	6.7 ± 3.21	0.88 ± 3.11	0.06
**Texture**	7.05 ± 2.79	6.59 ± 2.36	0.45 ± 2.68	0.26
**Irritation**	1.2 ± 0.63	1.57 ± 1.43	−0.36 ± 1.29	0.06
**Absortion Rate**	6.91 ± 2.28	6.55 ± 2.34	0.36 ± 2.47	0.33
**Application**	7.57 ± 2.65	7.66 ± 2.58	−0.09 ± 2.7	0.82
**Increases Skin Conditions**	7.82 ± 2.00	7.7 ± 1.72	0.11 ± 1.64	0.64
**Easy to Use**	8.7 ± 2.25	8.55 ± 1.91	0.15 ± 1.81	0.56
**Global Evaluation**	8.32 ± 1.77	7.8 ± 1.73	0.52 ± 1.95	0.08

The values for EVOO and petrolatum are calculated as the mean and standard deviation (±). Bold indicates statistically significant *p*-values (*p* < 0.05).

## Data Availability

The data presented in this study are available from the corresponding author on reasonable request.

## Abbreviations

The following abbreviations are used in this manuscript:

AU	Arbitrary units
C1C	Class 1 cells
C2C	Class 2 cells
C3C	Class 3 cells
C4C	Class 4 cells
C5C	Class 5 cells
EVOO	Extra virgin olive oil
TEWL	Transepidermal water loss
OO	Olive oil
SBF	Skin barrier function
SC	Stratum corneum
SCH	Stratum corneum hydration
